# Pediatric infection-triggered encephalopathy syndromes: a *multidimensional* biomarker analysis

**DOI:** 10.3389/fneur.2026.1730057

**Published:** 2026-03-18

**Authors:** Caihui Ma, Shuowen Wang, Zhijie Gao, Zijun Wang, Hui Jiao, Yizhi Zhang, Shuo Miao, Zhao Liu, Jianzhao Zhang

**Affiliations:** 1 Department of Neurology, Capital Center for Children's Health, Capital Medical University, Beijing, China; 2 Capital Center for Children’s Health, Capital Medical University, Beijing, China; 3 Beijing Shijitan Hospital, Capital Medical University, Beijing, China; 4 Department of Pharmacy, Capital Center for Children's Health, Capital Medical University, Beijing, China

**Keywords:** blood metabolites, cytokines, immune cells, infection-triggered encephalopathy syndrome (ITES), urinary metabolites

## Abstract

**Background:**

Pediatric infection-triggered encephalopathy syndromes (ITES) cause severe neurologic and cognitive deficits, but reliable biomarkers for early diagnosis and improved outcomes are lacking.

**Methods:**

This retrospective study analyzed the clinical characteristics and laboratory data from 48 children with infection-triggered encephalopathy syndromes, using a case–control design. Ultra-high-performance liquid chromatography–tandem mass spectrometry, the Luminex xMAP^®^ multiplex assay system, the Cobas^®^ 8,000 analyzer, and immunoturbidimetry were utilized to measure blood and urine metabolites, cerebrospinal fluid and plasma cytokines, and cerebrospinal fluid biomarkers and proteins.

**Results:**

Initial urinary metabolomic profiling identified 56 differentially abundant metabolites in the infection-triggered encephalopathy syndromes group (50 upregulated, 6 downregulated). Partial least-squares discriminant analysis highlighted 13 metabolites with variable importance in projection scores >1, 12 of which may serve as candidate biomarkers (area under the curve > 0.75; e.g., 3-hydroxybutyrate, fucose). Random Forest modeling prioritized five urinary metabolites: stearate, malate, glucose1, glucose2, and fucose. Similarly, five metabolites, such as C4OH, C14OH(CIL), C18:1OH, C10:2(CIL), and C5DC(CIL)/C16, may serve as potential biomarkers (AUC > 0.75). Cerebrospinal fluid analysis showed elevated interleukin-6 and interleukin-8 levels in the infection-triggered encephalopathy syndromes group (area under the curve > 0.75 each). Clinically, there were significant differences between the ITES group and the control group in terms of Modified Rankin Scale scores, infection status, fever, seizures, and altered consciousness (all *p* < 0.05).

**Interpretation:**

This study identifies a panel of urinary, plasma, and cerebrospinal fluid biomarkers, which provide a thorough molecular profile of infection-triggered encephalopathy syndromes in children. These findings provide a direction for future research on mechanistic studies, early identification, and risk classification.

## 1 Introduction

Infection-triggered encephalopathy syndromes (ITES) represent a spectrum of neuroinflammatory reactions that originate after common pediatric infections. In 2025, experts from Asia, Europe, and Oceania developed the International Consensus Definitions for ITES (1). The classification consists of:

1 Acute encephalopathy with biphasic seizures and late reduced diffusion (AESD).
2 Acute necrotizing encephalopathy (ANE).
3 Mild encephalopathy with reversible splenial lesion (MERS).
4 Acute fulminant cerebral oedema (AFCE).
5 Acute shock with encephalopathy and multiorgan failure (ASEM) (formerly “hemorrhagic shock and encephalopathy syndrome”) (1).

ITES frequently present with encephalopathy and seizures, reflecting widespread brain dysfunction. Less common symptoms include focal neurological deficits, such as hemiplegia. A clinical overlap of neurological symptoms with infectious and autoimmune encephalitis makes diagnosis challenging.

ITES occur in all age groups; nonetheless, children are more susceptible than adults, except for MERS (2, 3). Each subtype has an age distribution that facilitates diagnosis. Viral pathogens are the most common causes, including the influenza virus, human herpesvirus-6, respiratory syncytial virus, rotavirus, dengue virus, and severe acute respiratory syndrome coronavirus 2 (SARS-CoV-2) (2–5); however, bacterial infections (pathogenic *Escherichia coli* and acute focal bacterial nephritis) have also been associated (6, 7).

Pathological examinations suggest no presence of virus in the brain. Furthermore, cerebrospinal fluid (CSF) viral polymerase chain reaction (PCR) results are typically negative (8). Most ANE and AESD cases are marked by an absence of CSF pleocytosis, although it may be slightly elevated in febrile infection-related epilepsy syndrome and sporadically in MERS. Substantial CSF pleocytosis suggests primary encephalitis or meningitis. However, growing evidence indicates that neuroinflammation is central to ITES pathogenesis. The inflammatory response stems from an indirect immune response caused by systemic infections, which subsequently activates immune cells within the central nervous system, particularly microglia and astrocytes (9). Markers of neuroinflammation and excitotoxicity, such as neopterin and quinolinic acid, are more effective than CSF pleocytosis (10). They distinguish ITES and encephalitis from other factors leading to new-onset seizures or status epilepticus (10). Pro-inflammatory cytokines, including interleukin-6 (IL-6), are elevated in ITES (11). Anakinra, an antagonist of the interleukin-1 receptor, along with tocilizumab, which targets the IL-6 receptor, may hold promise (12, 13). Moreover, some familial cases of ANE are related to pathogenic mutations in the RANBP2, RNH1, or DBR1 genes (14–16). Additionally, particular human leukocyte antigens are associated with an increased risk of atypical immune reactions in ANE (17).

Previously, ITES had not been widely recognized internationally, which hindered research progress. No validated early biomarkers are available. Considering the hyperacute progression of ITES, early diagnosis is crucial. Neuroimaging is a key diagnostic method. Computed tomography (CT) scans are used for ASEM, AFCE, and ANE, whereas magnetic resonance imaging (MRI) is used for AESD/MERS. However, these methods detect abnormalities only after brain damage. Therefore, this study set out to identify earlier diagnostic markers for ITES by combining metabolic and immune profiling in children with ITES.

Pediatric ITES cases from the Children’s Hospital of Capital Medical University between December 2019 and February 2025 were retrospectively analyzed utilizing clinical data along with analyses of serum immune cells, cytokines in blood and CSF, urinary metabolites, and blood metabolites. Statistical methods were employed to identify key indicators with high diagnostic accuracy, thereby laying the foundation for the development of an early diagnostic model. This integrative strategy may greatly enhance early ITES detection, facilitate timely interventions, and lower the chances of long-term neurological complications.

## 2 Materials and methods

### 2.1 Cohort

The ITES group comprised children who were hospitalized at the Children’s Hospital of Capital Medical University between December 2019 and February 2025. The control group comprised age-matched and healthy children during the same period. Blood, urine, and CSF samples were collected from all participants. This study was approved by the Ethics Committee of the Children’s Hospital of Capital Medical University (SHERLL2025034).

The diagnostic criteria for ITES were as follows:

- Mode of onset: A febrile illness occurring within 1 week before the onset of neurological symptoms (typically occurring 1 to 2 days earlier), with fever commonly evident during neurological symptom onset.
- Clinical features: Encephalopathy, with or without other neurological symptoms.
- Neuroradiological and other investigative criteria.
- A thorough exclusion of other potential causes.

### 2.2 Targeted UPLC–MS/MS metabolite analysis

Targeted metabolomic analysis was conducted using an ACQUITY ultra-performance liquid chromatography–tandem mass spectrometry system Xevo TQ-S system, leveraging 310 reference standards (Sigma-Aldrich, Steraloids, TRC Chemicals). To ensure the metabolic integrity of the analytes, we recorded the exact pre-centrifugation clotting time (≤45 min at 2–8 °C), the total frozen-storage interval of each plasma aliquot (−80 °C, ≤6 months between collection and analysis), and the number of freeze–thaw cycles (maximum one, verified by electronic sample-logging). Any aliquot deviating from these criteria was excluded to minimize pre-analytical variance. Urine and serum samples were thawed on ice, lyophilized, and reconstituted in 50% methanol. To precipitate proteins, 120 μL of ice-cold methanol containing internal standards was added to each sample, followed by 5 min of vortexing and centrifugation at 4,000 × *g* for 30 min. The supernatant (30 μL) was derivatized with fresh reagents at 30 °C for 1 h, diluted with 50% ice-cold methanol, and centrifuged again at 4,000 × g for 30 min at 4 °C. The separation process utilized an ACQUITY UPLC BEH C18 column (1.7 μm, 2.1 × 100 mm) at 40 °C with a gradient elution of 0.1% formic acid (A) and a mixture of acetonitrile/isopropanol (70:30, B) at a flow rate of 0.40 mL/min over 18 min. Mass spectrometry operated in dual ionization modes (negative: 2.0 kV; positive: 1.5 kV), with source and desolvation temperatures set to 150 °C and 550 °C, respectively.

### 2.3 Univariate analysis

First, raw data were adjusted to correct for systematic differences among samples. Median normalization was applied to eliminate batch effects and improve comparability across samples. Second, a base-10 log transformation was conducted to standardize and normalize the data, thereby improving subsequent modeling. The data distribution was assessed for normality and resembled a Gaussian distribution, ensuring the appropriateness of parametric tests.

Potential biomarkers were detected using independent t-tests with significance thresholds of a false discovery rate (FDR) < 0.05 and a |log2[fold change]| > 1. In cases containing a high number of biomarkers, partial least squares discriminant analysis (PLS-DA) was utilized for dimensionality reduction. This technique reduced data complexity and helped identify the biomarkers that most significantly contributed to group separation.

### 2.4 Random forest model

Filtered metabolomic data were analyzed using the Random Forest machine learning algorithm, implemented via the randomForest package in R. All data were used to train the model. The parameters were set as follows: the number of trees (n tree) was set to 500, variables considered for each split (mtry) equaled the square root of the number of metabolites, the minimum size for nodes (nodesize) was set to 1, and the proximity parameter was activated to assess sample similarities. To evaluate the model’s performance, overall accuracy, group-specific error rates, and the receiver operating characteristic (ROC) and area under the ROC curve (AUC) were calculated using the pROC package.

Feature importance was ranked based on the MeanDecreaseGini metric, which enabled the identification of metabolites. To illustrate their relationships, a correlation matrix was created and visualized as a heatmap. Sample similarities were visualized using the proximity matrix, and a comparison between predicted and actual classifications was conducted using a confusion matrix, shown as a heatmap.

### 2.5 CSF and blood biomarker testing

Cerebrospinal fluid and matched blood samples were collected under aseptic conditions. CSF was obtained via a lumbar puncture, immediately aliquoted into sterile containers, and kept on ice. Blood samples were centrifuged at 1,500 × *g* for 10 min at 4 °C to isolate plasma. All samples were transported to the hospital laboratory within 30 min under refrigerated conditions (2–8 °C). Biochemical parameters of CSF comprised the total protein, glucose, and lactate. They were analyzed using a Cobas^®^ 8,000 analyzer (Roche Diagnostics). Albumin and immunoglobulin G (IgG) were quantified via immunoturbidimetry (Siemens ADVIA^®^ 2,400). Routine CSF testing included manual leukocyte counts with a Fuchs-Rosenthal chamber and differential cytospin staining using May-Grünwald-Giemsa. Cytokine levels in CSF and plasma were measured using a Luminex xMAP^®^ multiplex assay (Human Cytokine/Chemokine Panel, MilliporeSigma) on a Luminex® 200™ system. Calibration curves were plotted daily, and internal quality controls were implemented. Data were extracted from laboratory information systems, and any hemolyzed or clotted specimens were excluded.

### 2.6 Statistical analysis

All statistical analyses were conducted using R (version 4.3.2, The R Foundation for Statistical Computing). Chi-square tests were used to compare frequencies of categorical variables across groups. Continuous variables were normalized using the median and were log-transformed (base 10) to meet a Gaussian distribution. Additionally, two-tailed Student’s t-tests were applied for comparisons between the groups. Dimensionality reduction was attained through PLS-DA and random forest models to identify the most significant biomarkers. A *p*-value <0.05 was considered significant.

## 3 Results

### 3.1 Study cohort

This study included 48 children in the ITES group and 43 children in the control group. No significant differences in age and sex were observed between these groups. Metabolic and immunological assessments were conducted on specific participant subsets:

- Blood metabolomics: 20 children in the ITES group and 34 in the control group.
- Urine metabolomics: 18 children in the ITES group and 34 in the control group.
- Lymphocyte counts: 43 children in the ITES group and 36 children in the control group.
- Blood cytokine levels: 36 children in the ITES group and 23 in the control group.
- CSF cytokine levels: 24 children in the ITES group and 15 in the control group (Table 1).
- Pathogen spectrum: The most common pathogens in the ITES group included the influenza A virus (*n* = 17) and SARS-CoV-2 (*n* = 11). Other pathogens included the influenza B virus, human enterovirus, adenovirus, rotavirus, norovirus, Epstein–Barr virus, and *Mycoplasma pneumoniae*.
- CSF analysis: The average levels of CSF protein were higher in the ITES group than in the control group (298.8 g/L vs. 232.8 g/L). Two patients with ITES showed protein levels exceeding 10,000 mg/L, and both died. The average CSF glucose content was marginally higher in the ITES group than in the control group (3.6 mmoL/L vs. 3.1 mmoL/L); however, the difference was insignificant. CSF IgG levels were lower in the ITES group than in the control group (8.0 g/L vs. 9.9 g/L).
- Hematologic observations: Ten boys with ITES had platelet counts below 100 × 10^9/L, and only two of them survived.
- Clinical Results: The median length of hospitalization was 7.0 days in the ITES group. The average modified Rankin Scale (mRS) score was 2.4 ± 2.8 during discharge in the ITES group. This score reflects a significant difference in prognosis.
- Neuroimaging findings: All 48 patients in the ITES group underwent either cranial MRI or CT. Lesions were observed in the following areas: bilateral thalamus (25 cases), corpus callosum (8 cases), brainstem (17 cases), cerebellar hemispheres (15 cases), basal ganglia (9 cases), and white matter (10 cases) (Table 2).

**Table 1:** Sample size of the ITES group and control group.

Group	Number	Blood metabolites	Urinary metabolites	Lymphocyte	Blood cell cytokines	Cerebrospinal fluid cytokines
ITES group	48	20	18	43	36	25
Control group	43	34	34	36	23	15

**Table 2:** Comparison of some clinical characteristics between the ITES group and control group.

Variate	ITES group	Control group
Number	48	43
Pathogeny		
Influenza A	17	
Influenza B	2	
COVID-19	11	
Human rhinoenterovirus	2	
Adenovirus + rotavirus	1	
Norovirus	1	
Influenza A + Influenza B	1	
Influenza A + Mycoplasma	2	
Adenovirus +EB virus	1	
MRS (*^−^x* ± s)	2.4 ± 2.8	
Length of hospital stay (days) (range)	7.0 (1.0, 28)	
Cerebrospinal fluid white blood cells (×10^6^/l)	0 (0, 16)	0 (0, 3)
Cerebrospinal fluid protein (g/l)	298.8 (125.2, 13573.0)	232.8 (183.6, 398.8)
Cerebrospinal fluid glucose (mmol/l)	3.6 (2.9, 6.4)	3.1 (2.8, 3.8)
IgG (g/l)	8.0 (2.1, 32.9)	9.9 (6.1, 14.3)
Platelets (×10^9^/l)	138 (7, 330)	

ITES group: Infection-Triggered Encephalopathy Syndromes; Control group: children diagnosed with psychogenic physiological dysfunction; MRS: The Modified Rankin Scale (mRS) is a standardized clinician-reported measure of global functional outcomes in patients with stroke and other neurological disorders. The Modified Rankin Scale assesses the degree of disability after acute neurological injury. It ranges from 0 (no symptoms) to 6 (death) and is widely used to: Evaluate recovery in stroke clinical trials; Predict long-term functional independence Guide rehabilitation planning.

### 3.2 Univariate analysis of urine metabolite differences between ITES and control groups

**Figure 1:**
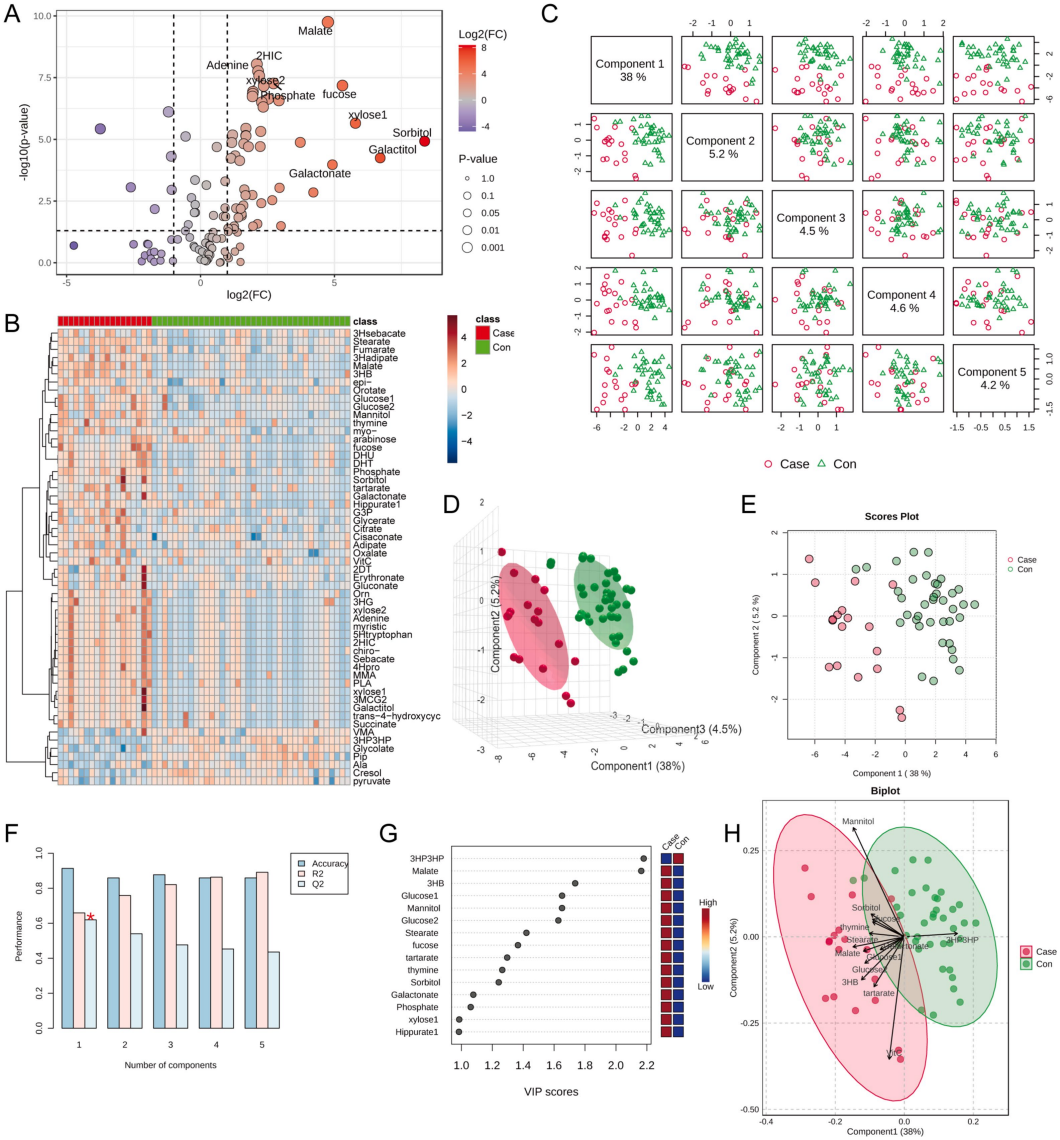
Global differences in urine metabolites between the control group (*n* = 34) and the ITES group (*n* = 18). **(A)** Volcano plot showing differentially abundant metabolites. Red and blue dots represent 50 upregulated and 6 downregulated metabolites, respectively (FDR < 0.05, |log_2_FC| > 1). **(B)** Bar plot showing the distribution of significantly altered metabolites. **(C)** Explained variance of top five principal components in the PLS-DA model (PC1: 38%). **(D)** Three-dimensional scores plot demonstrating group separation along PC1, PC2, and PC3 with minimal intra-group variation. **(E)** Two-dimensional scores plot highlighting inter-group discrimination by PC1 and PC2. **(F)** Cross-validation plot indicating significant predictive power for PC1 (*Q*^2^ > 0). **(G)** VIP scores for PC1. Thirteen metabolites with VIP > 1 are identified as key discriminators. **(H)** Biplot illustrating the contribution of VIP-selected metabolites to group separation. ITES, infection-triggered encephalopathy syndromes; FC, fold change; FDR, false discovery rate; PLS-DA, partial least squares–discriminant analysis; *Q*^2^, cross-validated predictivity; PC, principal component; and VIP, variable importance in projection.

First, urine metabolite data were normalized by median and log-transformed to minimize biases and better approximate a normal distribution (Supplementary Figure S1). Comparisons between the ITES and control groups were conducted using a significance threshold of [FDR] < 0.05 and |log2[FC]| > 1. This analysis yielded 56 differential urine metabolites, including 50 upregulated and 6 downregulated metabolites (Figures 1A,B). Subsequently, to refine the data and identify differential metabolites, PLS-DA was conducted. In the pairwise score plot, Principal Component 1 (PC1) accounted for the highest overall variance (38%) (Figure 1C). Three-dimensional plots, including PC1, PC2, and PC3, showed distinct inter-group differences, with minimal intra-group variations (Figure 1D). This trend was also observed in the two-dimensional scores plot of PC1 and PC2 (Figure 1E). Furthermore, the Q2 value for PC1 was significant, indicating its high predictive accuracy for classifying new data (Figure 1F). Variable Importance in Projection (VIP) values for PC1 yielded 13 metabolites with VIP values >1, suggesting significant contributions to the model’s classification performance (Figure 1G). Finally, a Biplot further illustrated the relationship between these 13 metabolites and sample classification (Figure 1H).

**Figure 2:**
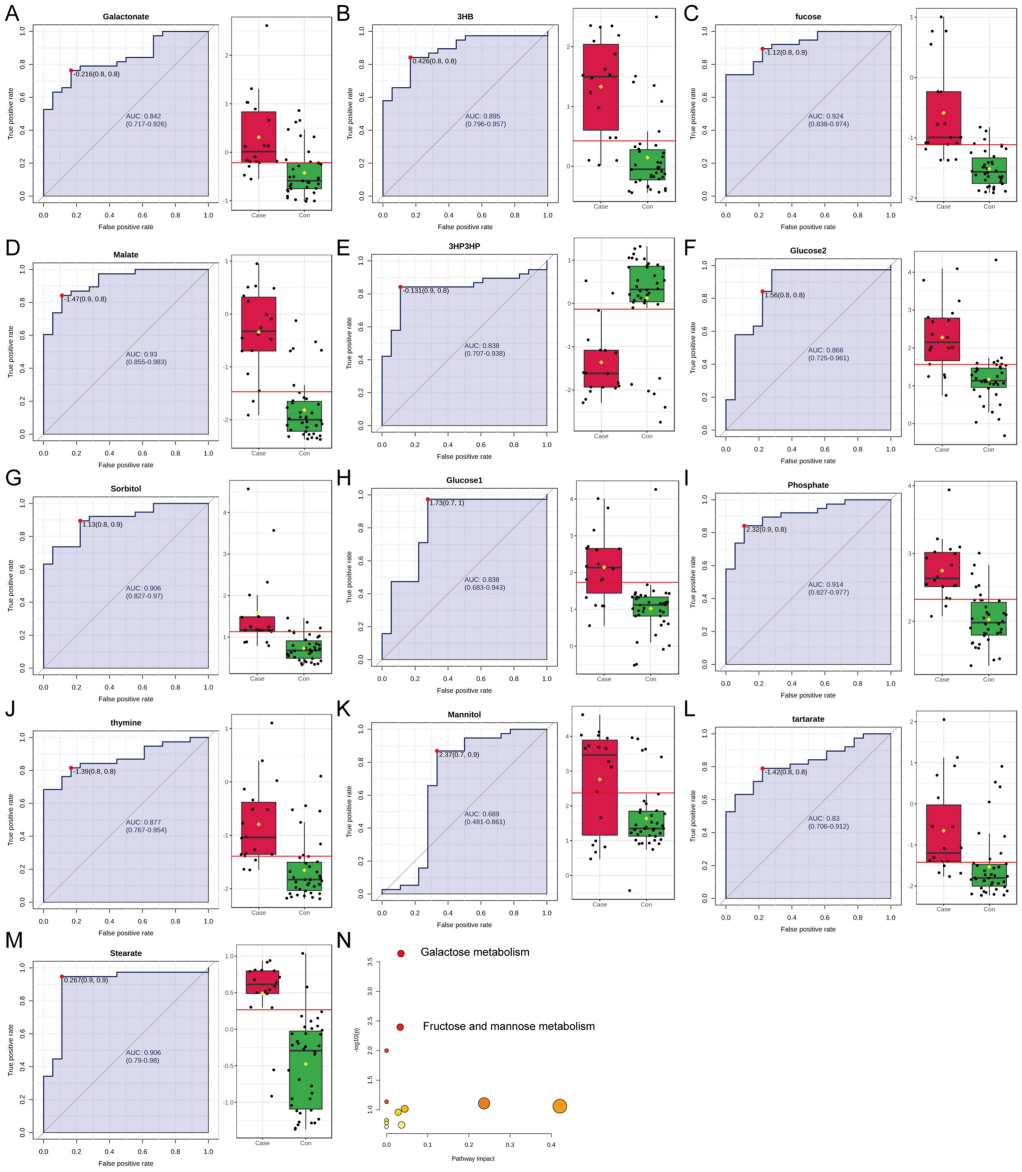
Biomarker validation and metabolic pathway analysis between the control group (*n* = 34) and the ITES group (*n* = 18). **(A–M)** ROC curves for 13 candidate biomarkers with an AUC > 0.75. Each subplot demonstrates the classification performance for: **(A)** galactonate, **(B)** 3HB, **(C)** fucose, **(D)** malate, **(E)** 3HP3HP, **(F)** glucose2, **(G)** sorbitol, **(H)** glucose1, **(I)** phosphate, **(J)** thymine, **(K)** mannitol, **(L)** tartarate, and **(M)** stearate. **(N)** KEGG pathway enrichment analysis showing significant alterations in galactose metabolism and fructose/mannose metabolism pathways (FDR < 0.05). ROC, receiver operating characteristic; ITES, infection-triggered encephalopathy syndromes; AUC, area under the ROC curve; KEGG, Kyoto Encyclopedia of Genes and Genomes; and FDR, false discovery rate.

In summary, the selected principal components, particularly PC1, effectively captured inter-group differences, and these 13 metabolites with significant contributions may serve as key candidates for biomarker research. The classification effectiveness of each metabolite was assessed through the ROC curve analysis. An AUC threshold >0.75 was established to detect metabolites that may serve as potential biomarkers between the ITES and control groups. Twelve metabolites (3HB, Fucose, Galactonate, 3HP3HP, Glucose2, Malate, Glucose1, Phosphate, Sorbitol, Tartarate, Thymine, and Stearate) may serve as candidate biomarkers for distinguishing the ITES and CON groups (Figures 2A–M). Kyoto Encyclopedia of Genes and Genomes (KEGG) pathway enrichment analysis showed that these differentially expressed metabolites were predominantly related to carbohydrate metabolism: (i) galactose metabolism and (ii) fructose and mannose metabolism (Figure 2N).

### 3.3 Construction of a random forest model for urine metabolites

**Figure 3:**
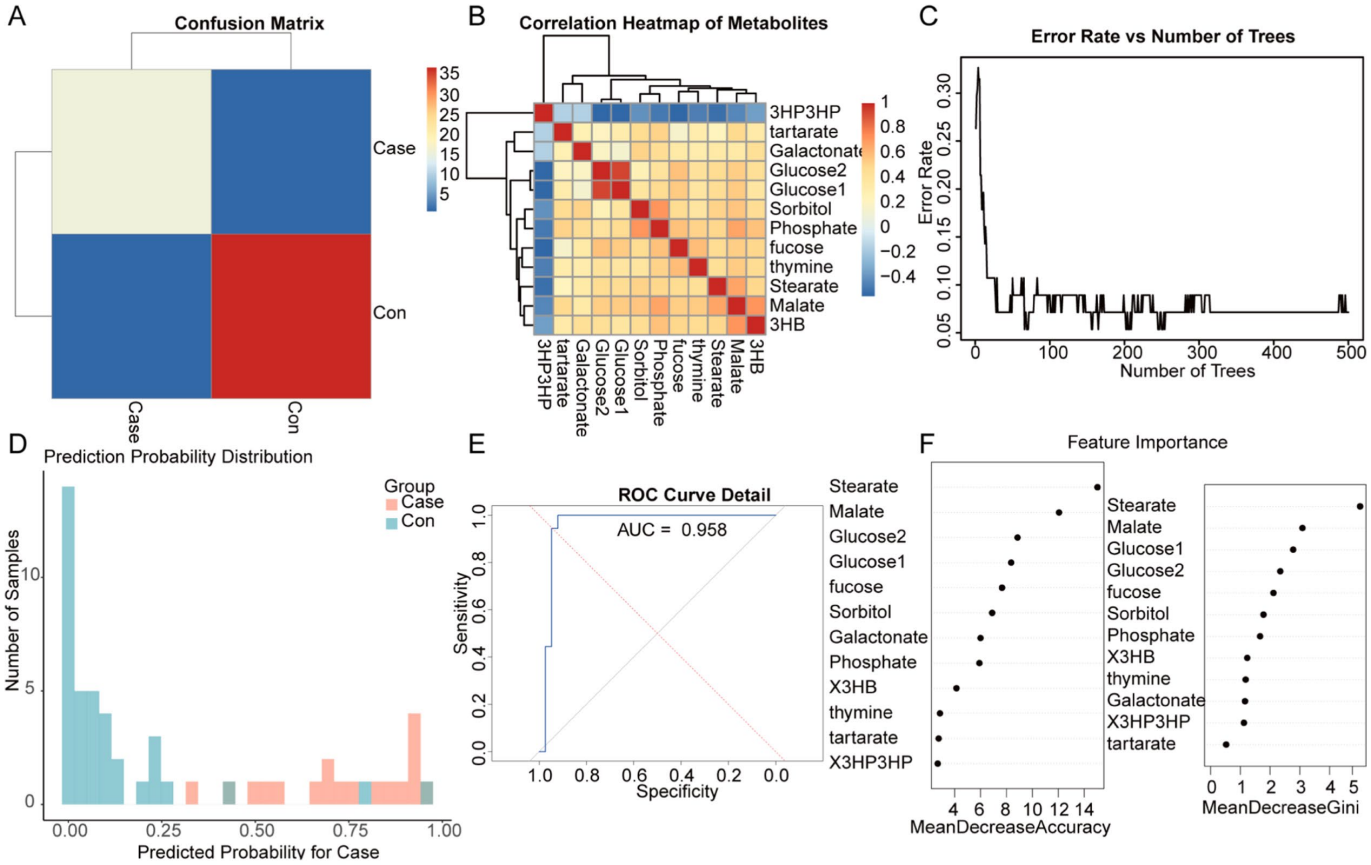
Random forest classification of urinary metabolites between the control group (*n* = 34) and the ITES group (*n* = 18). **(A)** Confusion matrix heatmap showing high concordance between predicted and actual group classification. **(B)** Correlation heatmap of differentially abundant metabolites. Red and blue indicate positive and negative correlations, respectively. **(C)** OOB error rate against the number of decision trees. **(D)** Prediction probability distributions for ITES (red) and CON (blue) groups, showing robust classification. **(E)** ROC curve of the random forest model. **(F)** Feature importance ranking, showing the top five discriminative metabolites: stearate, malate, glucose1, glucose2, and fucose. ITES, infection-triggered encephalopathy syndromes; ROC, receiver operating characteristic; and OOB, out-of-bag.

To further filter effective variables and mitigate the risk of overfitting, the random forest algorithm was utilized on the urinary metabolite dataset obtained from univariate analysis. The confusion matrix heatmap (Figure 3A) demonstrated a strong agreement between the predicted and actual group classifications, indicating high accuracy of the model. The correlation heatmap (Figure 3B) elucidates the complex relationships among urinary metabolites, highlighting clusters of highly correlated features that may indicate potential metabolic pathways or related biological processes.

The error rate per tree plot (Figure 3C) suggested a notable decrease in both overall and group-specific error rates with an increase in the number of trees, indicating that the model’s stability and reliability improve with the number of trees. This finding suggests enhanced generalizability and robustness of the model with larger ensemble sizes. The prediction probability distribution plot (Figure 3D) presents a distinct separation between the ITES and control groups, highlighting the model’s effectiveness in classification.

The ROC curve (Figure 3E) demonstrates the model’s robust diagnostic ability, with an AUC exceeding 0.75. The random forest model demonstrated exceptional classification performance, indicating a high level of accuracy in distinguishing between the groups. The feature importance plot (Figure 3F) suggested that five metabolites, namely stearate, malate, glucose1, glucose2, and fucose, are central to differentiating between the ITES and control groups. These metabolites may serve as potential biomarkers for ITES and warrant further investigation.

### 3.4 Univariate analysis of blood metabolite differences between ITES and control groups

**Figure 4:**
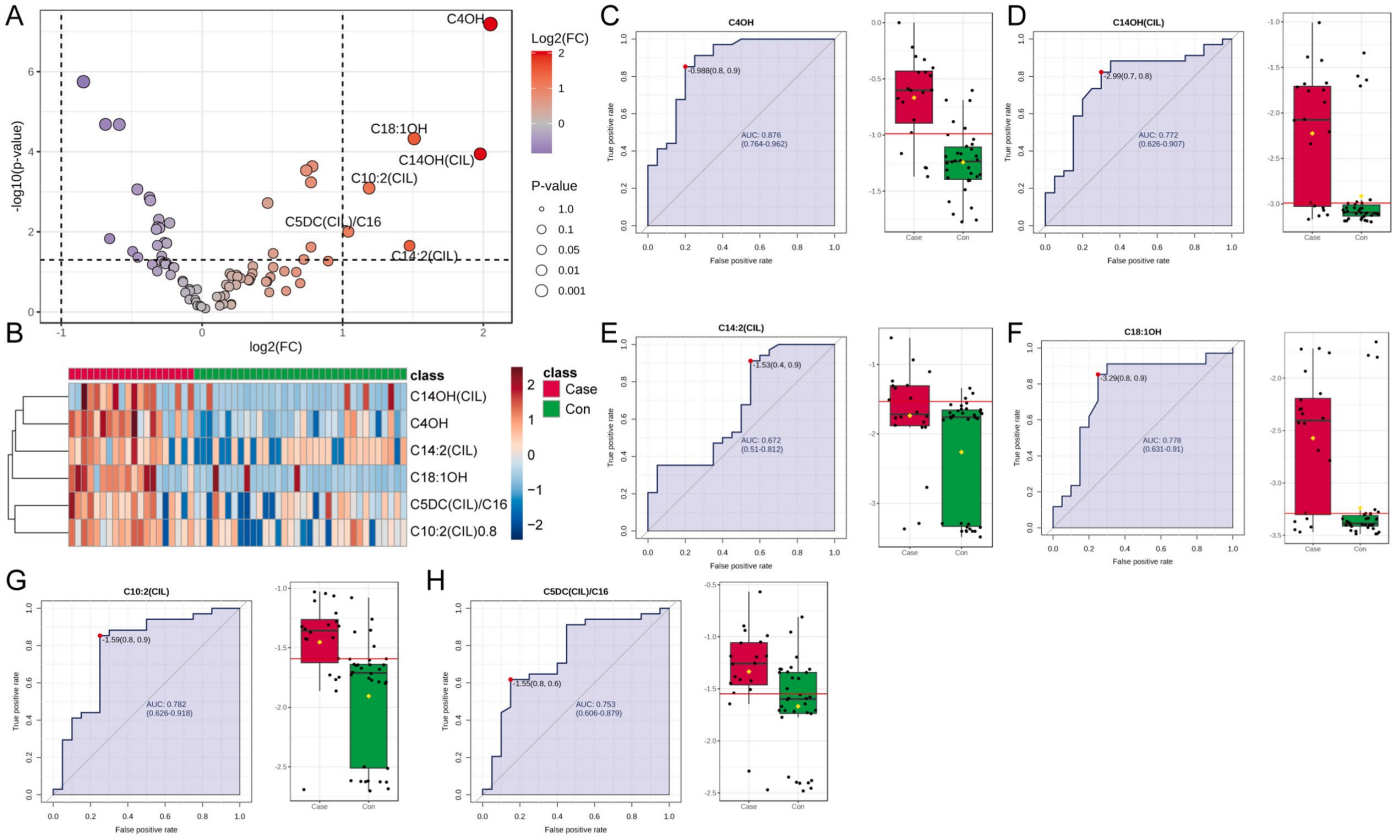
Differentially expressed metabolites in blood samples and their discriminative ability between the ITES (*n* = 20) and control groups (*n* = 34). **(A)** Volcano plot of differential metabolite screening (FDR < 0.05 and |log_2_(FC)| > 1). **(B)** Bar chart showing abundance changes [log_2_(FC)] for six significantly upregulated metabolites. **(C–H)** Relative expression levels, ROC curves, and AUC values for individual metabolites: C4OH **(C)**, C14OH(CIL) **(D)**, C14:2(CIL) **(E)**, C18:1OH **(F)**, C10:2(CIL) **(G)**, and C5DC(CIL)/C16 **(H)**. ITES, infection-triggered encephalopathy syndromes; FC, fold change; FDR, false discovery rate; ROC, receiver operating characteristic; and AUC, area under the ROC curve.

The blood metabolite data underwent similar processing using the same normalization techniques as the urinary dataset, which included median normalization and log transformation (Supplementary Figure S2). Statistical analysis applying a significance threshold of [FDR] < 0.05 and |log2[FC]| > 1 suggested six differentially expressed metabolites. All were significantly upregulated in the ITES group (Figures 4A,B). Through ROC curve analysis and an AUC threshold > 0.75, five metabolites—C4OH, C14OH(CIL), C18:1OH, C10:2(CIL), and C5DC(CIL)/C16—may serve as candidate biomarkers for distinguishing the ITES and control groups (Figures 4C–H). Hence, a subset of blood metabolites may serve as markers for ITES.

### 3.5 Construction of a random forest model for blood metabolites

**Figure 5:**
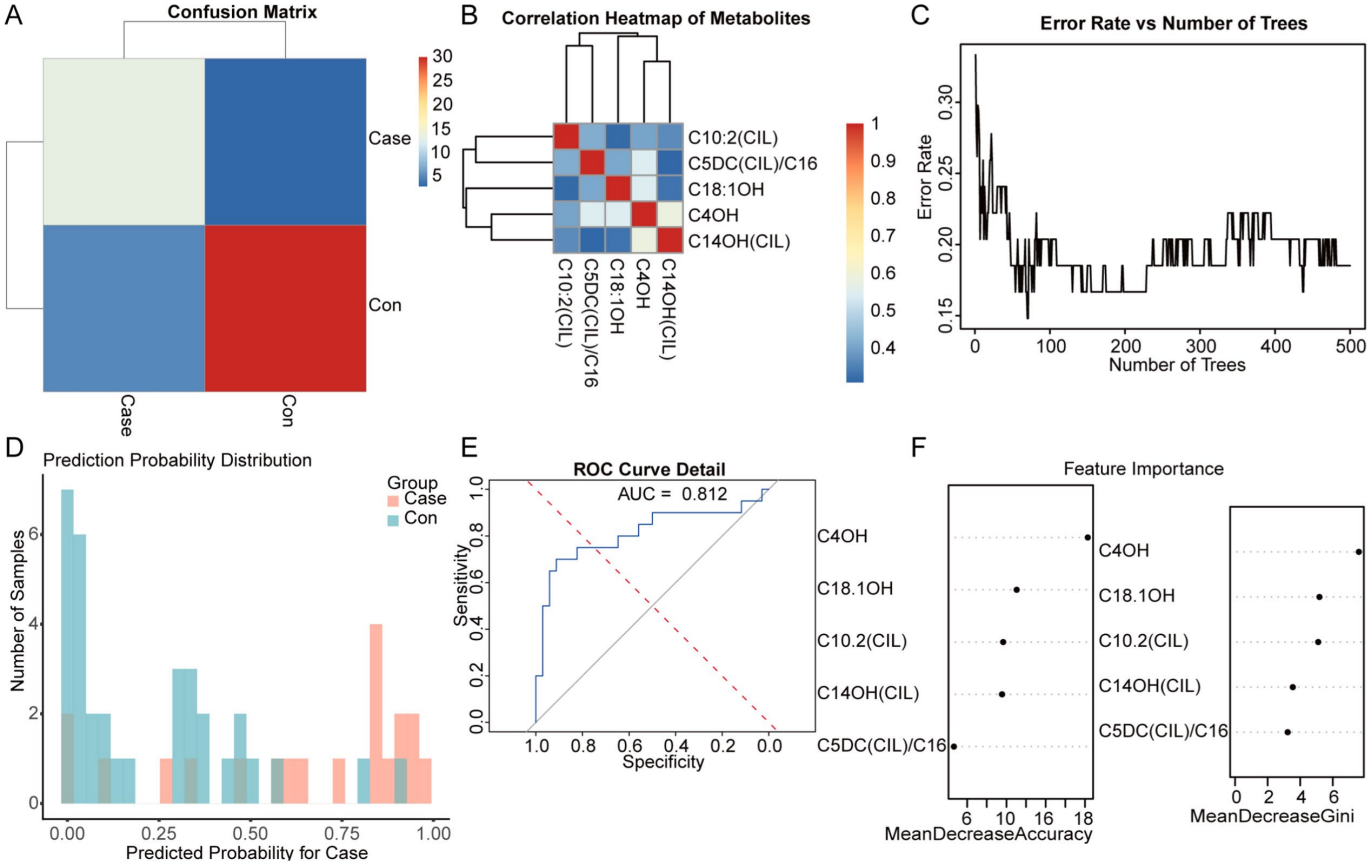
Machine learning validation of blood metabolite biomarkers for distinguishing the ITES group (*n* = 20) from the control group (*n* = 34). **(A)** Confusion matrix heatmap demonstrating high agreement between predicted and actual group classifications. **(B)** Correlation heatmap of blood metabolites. **(C)** OOB error rate against the number of decision trees, indicating enhanced model stability with larger ensemble size. **(D)** Prediction probability distributions showing separation between ITES and control groups. **(E)** ROC curve of the random forest model with AUC > 0.75, confirming robust diagnostic performance. **(F)** Feature importance ranking identifying C4OH, C14OH(CIL), C18:1OH, C10:2(CIL), and C5DC(CIL)/C16 as key metabolites. ITES, infection-triggered encephalopathy syndromes; OOB, out-of-bag; ROC, receiver operating characteristic; and AUC, area under the ROC curve.

The random forest modeling approach was similarly used for analyzing blood metabolites. The confusion matrix heatmap (Figure 5A) suggested a high degree of consistency between the predicted and actual classifications. The correlation heatmap (Figure 5B) indicated the relationships among blood metabolites, elucidating shared biochemical pathways. The plot showing error rate per tree (Figure 5C) indicated improved model stability with an increase in the number of trees. In contrast, the distribution of prediction probabilities showed a distinct separation between the two groups (Figure 5D). The ROC curve (Figure 5E) with an AUC > 0.75 confirmed the model’s robust predictive accuracy and reliability. Feature importance plot (Figure 5F) identified C4OH, C14OH(CIL), C18:1OH, C10:2(CIL), and C5DC(CIL)/C16 as the most significant variables, highlighting their role in differentiating between groups.

### 3.6 Univariate analysis of blood and CSF biomarker differences between ITES and control groups

**Figure 6:**
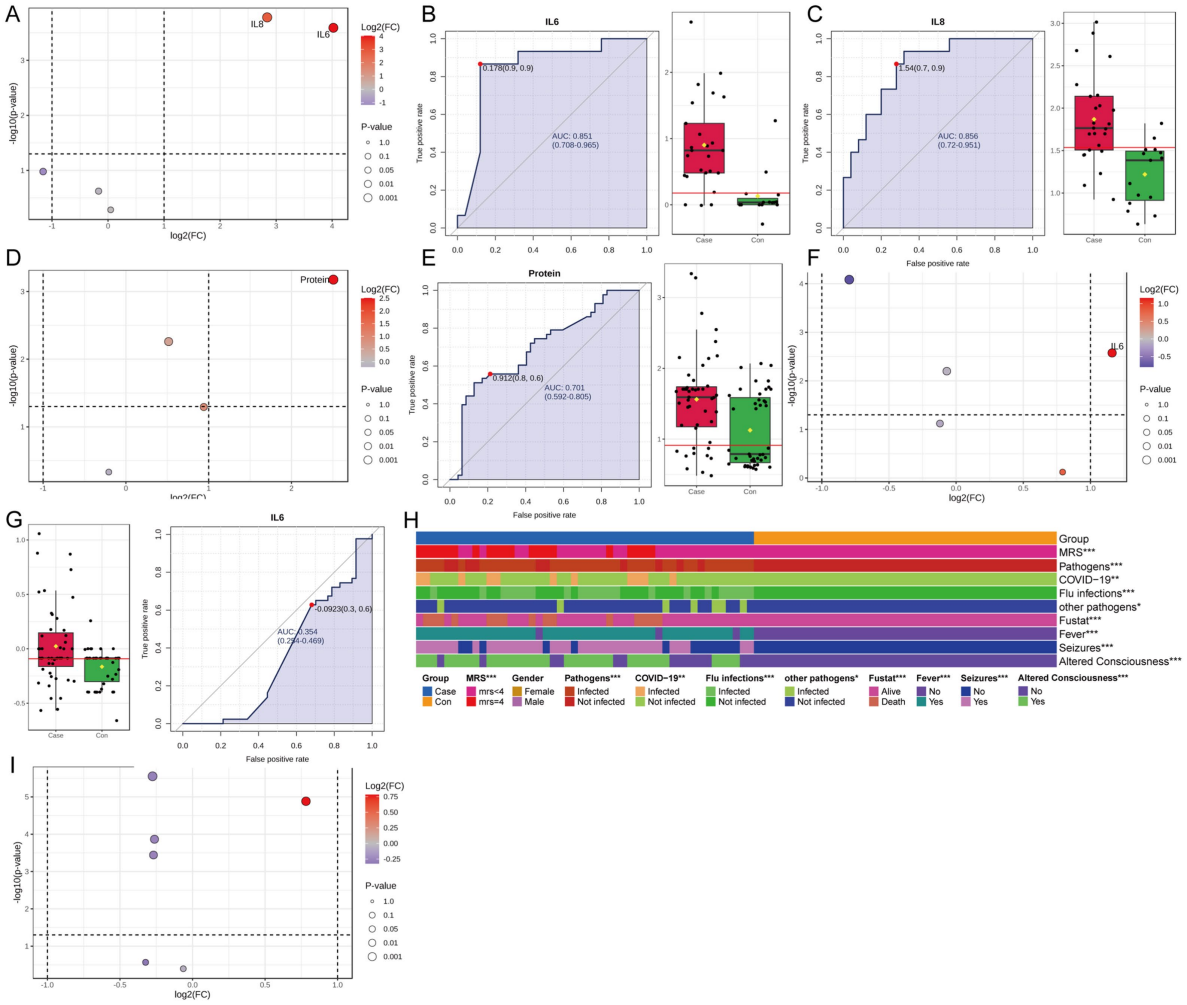
Biomarker and clinical analyses in CSF and blood distinguishing the ITES group from the control group. **(A–C)** CSF cytokine analysis between the control group (*n* = 15) and the ITES group (*n* = 25). **(A)** Volcano plot showing differential metabolites (FDR < 0.05 and |log_2_(FC)| > 1). **(B,C)** IL-6 and IL-8 levels are elevated in the ITES group (*p* < 0.05), with ROC curves showing excellent discriminative capacity (AUC > 0.75 for both cytokines). **(D,E)** CSF biochemical analysis between the control group (*n* = 15) and the ITES group (*n* = 25). **(D)** Volcano plot showing differential metabolites (FDR < 0.05 and |log_2_(FC)| > 1). **(E)** ROC curve for total protein showing poor diagnostic efficacy, with an AUC < 0.75. **(F,G)** Blood cytokine analysis between the control group (*n* = 23) and the ITES group (*n* = 36). **(F)** Volcano plot showing differential metabolites (FDR < 0.05 and |log_2_(FC)| > 1). **(G)** ROC curve for IL-6 indicating non-discriminative capacity (AUC < 0.75). **(I)** Immune cell profiling shows no significant intergroup differences. **(H)** Clinical characteristic heatmap: Chi-square test shows significant distribution differences (*p* < 0.05) in mRS score, age, pathogen type (COVID-19, influenza, or other), functional status (fustat), and clinical symptoms (fever, seizures, and altered consciousness). ITES, infection-triggered encephalopathy syndromes; CSF, cerebrospinal fluid; FDR, false discovery rate; FC, fold change; ROC, receiver operating characteristic; AUC, area under the ROC curve; mRS, modified Rankin Scale; and COVID-19, coronavirus disease 2019.

Considering the clinical implication of inflammatory and biochemical profiling in encephalitis, blood cytokines, blood immune cells, CSF cytokines, and CSF routine and biochemical parameters were assessed. To this end, the data were standardized, and a comparative analysis was conducted to assess the differences between the ITES and control groups (Supplementary Figure S3). In the CSF cytokine analysis, IL-6 and IL-8 levels were significantly higher in the ITES group, compared with the control group (Figures 6A–C). Each AUC was >0.75, demonstrating. Additionally, CSF protein levels were significantly elevated in the ITES group (Figures 6D,F). The AUC value was >0.7, indicating a good level of classification performance. In the blood cytokine analysis, IL-6 levels were significantly higher in the ITES group than in the control group (Figures 6E,F), however, its AUC was <0.5, suggesting limited ability to distinguish these groups. No significant differences were observed in the immune cell analysis (Figure 6G).

Finally, clinical characteristics were compared using the chi-square test. Significant differences were observed between the groups across various characteristics, including the mRS score, age, pathogen type, coronavirus disease 2019 status, influenza infection, infections from other pathogens, functional status (fustat), fever symptoms, seizures, and changes in consciousness (*p* < 0.05) (Figure 6H).

### 3.7 Subgroup analysis according to disease severity

**Figure 7:**
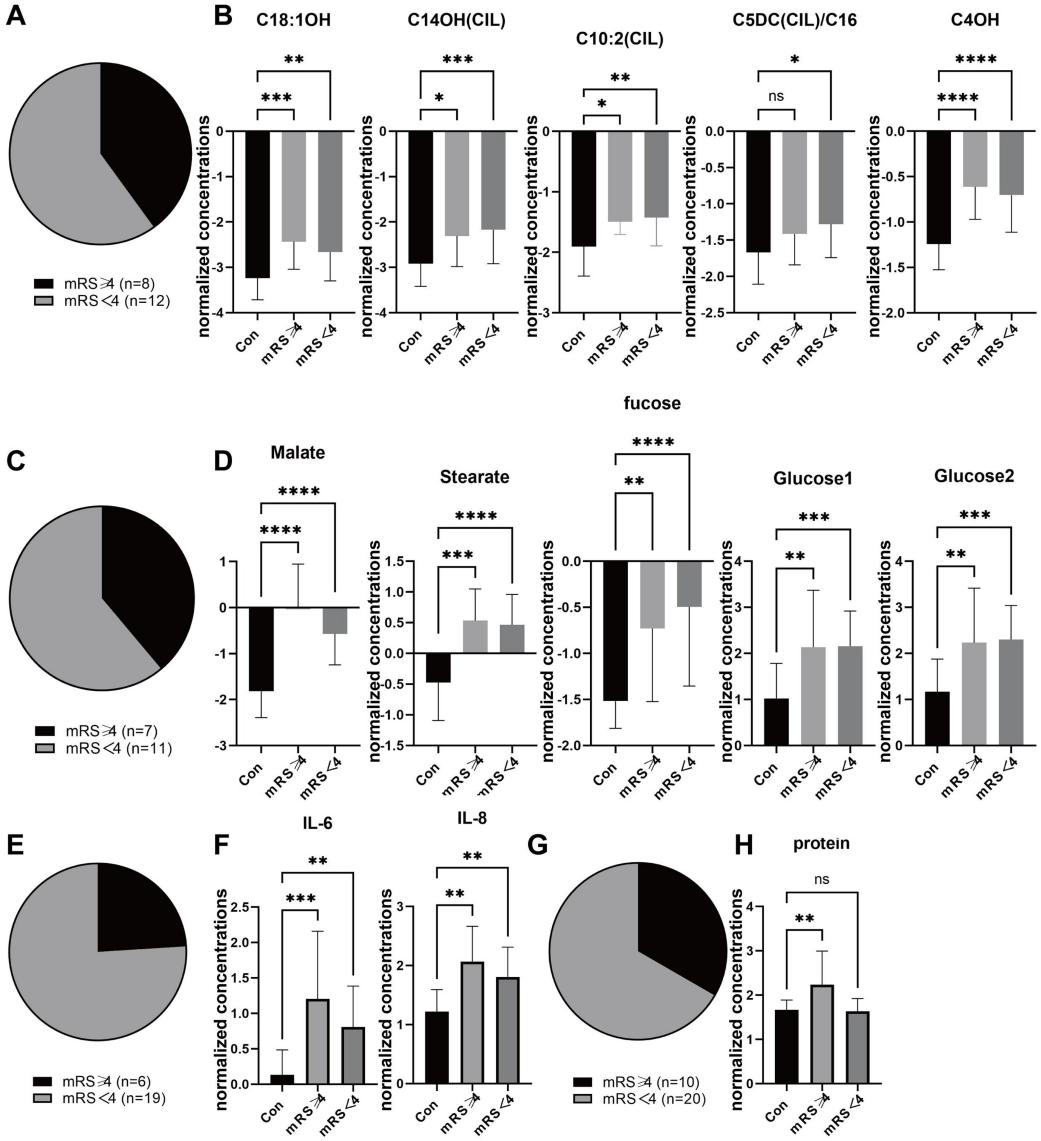
Sensitivity analysis of metabolic and CSF biomarkers stratified by functional outcome. Patients were stratified into poor outcome (mRS ≥ 4) and good outcome (mRS < 4) groups. **(A)** Distribution of mRS scores (*n* = 8 vs. *n* = 12) in blood metabolites. **(B)** Levels of blood metabolites including C4OH, C14OH(CIL), C18:1OH, C10:2(CIL), and C5DC(CIL)/C16. **(C)** Distribution of mRS scores in urine metabolites (*n* = 7 vs. *n* = 11). **(D)** Levels of urine metabolic intermediates including Malate, Stearate, Fucose, Glucose1, and Glucose2. **(E)** Distribution of mRS scores (*n* = 6 vs. *n* = 19) in CSF cytokines. **(F)** Levels of CSF cytokines IL-6 and IL-8. **(G)** Distribution of mRS scores (*n* = 10 vs. *n* = 20) in CSF protein. **(H)** Protein concentration. Data are presented as mean ± SD. Statistical significance was determined by one-way ANOVA or *t*-test. *p* < 0.05, *p* < 0.001, *p < 0.0001, ns = not significant.

To address the potential confounding effects of systemic illness severity and distinguish encephalopathy-specific metabolic alterations from general critical illness, we performed a sensitivity analysis by stratifying patients into two groups based on neurological outcomes at discharge: those with favorable outcomes (mRS < 4) and those with poor outcomes (mRS ≥ 4). The demographic distribution of patients with poor outcomes across different sample cohorts is illustrated in the pie charts (Figures 7A,C,E,G). As shown in Figures 7B,D, the magnitude of metabolic disturbances was significantly associated with disease severity. Specifically, the dysregulation of carnitine metabolism was more pronounced in patients with poor outcomes. Levels of long-chain hydroxyacylcarnitines, including C18:1OH, C14OH(CIL), and C16:2(OH)CL, as well as dicarboxylic acylcarnitines (C6DC-C16), were significantly decreased in the poor-outcome group compared to controls, while intermediate metabolites like Malate and Stearate were significantly elevated only in this severe subgroup. Furthermore, the severity of encephalopathy correlated strongly with the inflammatory profile (Figure 7F). Patients with poor outcomes exhibited significantly higher levels of CSF IL-6 and IL-8 compared to those with favorable outcomes, suggesting a pivotal role of the cytokine storm in severe neurological damage. Although CSF protein levels showed some elevation in severe cases, the difference did not reach statistical significance after correction for multiple testing (Figure 7H). Collectively, these findings indicate that the identified biomarkers, particularly the alterations in carnitine metabolism and neuroinflammation, are not merely reflective of general infection but are closely associated with the severity of neurological involvement in ITES.

### 3.8 Characterization of ITES subcomponents and their biomarker expression

The compositional analysis across biological samples revealed a consistent predominance of ANE, followed by MERS and possible ITES, with AESD being the least prevalent (Supplementary Figures S4A,C,E,G). Correspondingly, elevated expression trends of ITES subcomponents were observed in blood metabolites, urinary metabolites, and CSF cytokines compared to controls (Supplementary Figures S4B,D,F), whereas the expression profile in CSF proteins was less distinct (Supplementary Figure S4H).

## 4 Discussion

The ITES constitute a large group of conditions that often lead to concerning outcomes. Among these disorders, only MERS has a favorable prognosis, whereas the other clinico-radiological subtypes have been related to worse outcomes, such as epilepsy, neurodevelopmental issues, and motor disability (18). Despite growing awareness, ITES is still underdiagnosed globally and is often misdiagnosed as viral encephalitis, acute disseminated encephalomyelitis, or seronegative autoimmune encephalitis.

To date, there is no evidence-based therapy for ITES. Published reports primarily describe neuroprotective strategies, which include maintaining cerebral perfusion pressure, aggressive treatment of seizures, and regulating body temperature through normothermia or hypothermia (19, 20). Severe hyperinflammation provides a rationale for immune-targeted therapies (11, 21). Although there is a lack of high-quality evidence showing that immune therapy changes the disease course in ITES, anecdotal evidence suggests that early treatment with intravenous methylprednisolone may improve outcomes in severe ITES, such as ANE (22). Likewise, the early use of anti-cytokine therapies, such as tocilizumab and anakinra, is gaining interest, although more research is required to confirm their efficacy (12, 23, 24). Most reports suggest that earlier initiation of therapy may improve prognosis, emphasizing early ITES screening and diagnosis. Current diagnostic and therapeutic approaches primarily rely on clinical symptoms and imaging results, highlighting an urgent need for specific biomarkers and early diagnostic tools. The present study addresses this research gap by exploring metabolic and cytokine profiles in urine, blood, and CSF to identify ITES biomarkers.

Through targeted metabolomics, the metabolic profiles in the urinary and blood samples of patients with ITES were analyzed, comparing them with healthy children. In the urinary metabolite analysis, univariate statistical testing identified 56 candidate biomarkers with differing abundance. To avoid overfitting and prioritize the most representative variables, PLS-DA was utilized. It reduced the feature set down to 13 metabolites that exhibited significant differences between the groups.

Additional refinement using the AUC excluded biomarkers with insufficient discriminatory ability (AUC < 0.75), yielding 12 candidates. The Random Forest model suggested that five metabolites, stearate, malate, glucose1, glucose2, and fucose, had the highest clinical relevance. These metabolites appeared to distinguish the ITES group from the control group with notable differences, suggesting their potential as candidate diagnostic biomarkers that merit further investigation.

A similar analytical strategy was utilized for blood metabolites. First, univariate analysis identified six metabolites with significant differences between the ITES and control groups, with five surpassing the AUC threshold (>0.75). Thus, they were included in the Random Forest model. Finally, five potential biomarkers were identified: C4OH, C14OH(CIL), C18:1OH, C10:2(CIL), and C5DC(CIL)/C16. These results indicate that blood metabolites, in addition to urinary metabolites, can serve as potential biomarkers for ITES, providing a comprehensive biochemical fingerprint. Additionally, CSF analysis showed significantly increased IL-6 and IL-8 levels in the ITES group, each demonstrating strong discriminatory power (AUC > 0.75). To address potential confounding by systemic illness, we stratified ITES patients by mRS. This sensitivity analysis revealed that both metabolic and inflammatory disturbances were significantly more severe in patients with poor outcomes. Specifically, marked reductions in long-chain and dicarboxylic acylcarnitines, along with elevated CSF IL-6 and IL-8, were closely associated with the degree of neurological impairment. These findings suggest that the identified biomarkers reflect encephalopathy-specific pathophysiology rather than general critical illness. Given the recognized clinical heterogeneity of the ITES spectrum, an exploratory analysis of key biomarkers across major subtypes (e.g., ANE and MERS) was performed. This analysis revealed consistent directional changes in metabolic and inflammatory markers across subtypes compared to controls; however, the limited sample size within each subgroup precluded definitive statistical conclusions, highlighting the need for validation in larger, subtype-stratified cohorts.

Stearate, malate, glucose 1, glucose 2, and fucose were identified as the most discriminatory urinary markers for ITES, enriched in galactose and fructose/mannose metabolism pathways. Urinary glucose metabolites serve as surrogate indicators of systemic glycemic status, indirectly reflecting cerebral glucose delivery when blood glucose concentrations exceed renal tubular reabsorption capacity. Although direct evidence linking glucose metabolic disruption to ITES remains scarce, analogous perturbations in neurodegenerative conditions suggest that urinary glucose metabolites differentiate patients from healthy controls (25, 26), whereas elevated urinary fructose and altered succinate/pyruvate levels reflect oxidative stress, inflammation, and mitochondrial dysfunction (27). Systemic inflammation disrupts glucose homeostasis, compromising neuronal energy availability and ATP production (28), and transient, localized metabolic disruptions in white matter structures underlie reversible imaging changes during systemic stress (29). These findings address the knowledge gap regarding ITES pathophysiology, positioning urinary glucose metabolites as promising non-invasive diagnostic candidates (pending validation) and glucose metabolic modulation as a novel therapeutic strategy within the broader spectrum of neurological energy metabolism disorders.

The five blood metabolites identified as potential biomarkers—C4OH (3-hydroxybutyrylcarnitine), C14OH(CIL) and C18:1OH (hydroxy long-chain acylcarnitines), C10:2(CIL) (decadienylcarnitine), and the ratio C5DC(CIL)/C16 (glutarylcarnitine/palmitoylcarnitine)—all belong to the acylcarnitine family. Acylcarnitines are essential carriers for transporting fatty acids into mitochondria for *β*-oxidation, the primary process for cellular energy generation from lipids. Their accumulation in our ITES cohort, particularly the hydroxylated and dicarboxylic species, strongly suggests impaired mitochondrial fatty acid β-oxidation. Specifically, elevated C4OH indicates a shift toward ketogenesis and fatty acid utilization under stress. Increased hydroxylated long-chain acylcarnitines (C14OH, C18:1OH) are classic markers of incomplete fatty acid oxidation due to mitochondrial dysfunction. An altered C5DC/C16 ratio further reflects a disturbance in the balance between energy substrate metabolism and detoxification pathways. Notably, dysregulated acylcarnitine metabolism has been extensively documented across neurodegenerative conditions and cerebrovascular incidents, with these alterations implicated as biomarkers of disease progression, mitochondrial DNA integrity, and cognitive decline (30–35). Collectively, this acylcarnitine profile points to a state of systemic bioenergetic crisis and mitochondrial stress during ITES, where the immense energy demand of the brain conflicts with a compromised cellular energy production system. This metabolic dysfunction may not only contribute to neuronal injury but also represents a potential therapeutic target for metabolic support.

An analysis of CSF cytokines suggested that IL-6 and IL-8 levels were significantly elevated in the ITES group. The analysis of bloodstream cytokines suggested significantly increased IL-6 levels in the ITES group. However, the AUC was <0.5 for IL-6, indicating poor distinguishing ability between the ITES and control groups. IL-6 is a multifunctional cytokine that is secreted in substantial amounts after neural injury, particularly during infections, trauma, and neurodegenerative disorders. It promotes inflammation by activating glial cells (such as astrocytes and microglia), which may aggravate neural damage. IL-8, characterized by its strong chemoattractant properties for neutrophils and monocytes, amplifies the inflammatory response by promoting the recruitment of immune cells and activating localized inflammation. The roles of IL-6 and IL-8 are interconnected in neuroinflammation, both of which regulate neuroimmune processes and prolong neuroinflammation.

The AESD cases confirm these findings: case reports have shown elevated IL-6 levels in the CSF during the acute phase, suggesting a potential relationship between IL-6 levels and the inflammatory response in AESD (35). Despite treatment, these patients often experience sequelae, such as hemiplegia and intellectual impairment, indicating that persistently elevated IL-6 is associated with neurological outcomes. Mechanistically, IL-6 is a hallmark of “biphasic seizures” in AESD and the delayed restriction of cortical diffusion via blood–brain barrier disruption and neuroexcitotoxicity (36). Additionally, in influenza-associated encephalopathy, IL-6, IL-10, and TNF-*α* levels in the serum are considerably higher in patients who experience neurological complications. Furthermore, these levels are higher in fatal cases (37). The rise in CSF IL-6 and IL-8 levels in ITES suggests that inflammatory regulation primarily occurs in the central nervous system. This warrants investigating targeted anti-inflammatory pathways (such as IL-6 receptor antagonists or anti-IL-8 therapy) to reduce neuronal injuries.

Through the integration of urinary, blood, and CSF biomarker data, this study identified several metabolites and cytokines that exhibit strong discriminatory capabilities for ITES. The combination of these strategies enhances diagnostic accuracy, facilitates a better understanding of disease mechanisms, and provides a direction for future research on developing more effective diagnostic and therapeutic strategies. While our multimatrix approach provides a comprehensive view, it is important to stratify the clinical utility of these biomarkers. Urinary metabolites, particularly those related to glucose and galactose metabolism, demonstrated the highest diagnostic performance in our Random Forest models. Given the non-invasive nature of urine collection, these metabolites represent ideal candidates for early screening and risk stratification in pediatric populations. In contrast, while CSF cytokines (IL-6 and IL-8) require invasive procedures, they offer irreplaceable insights into the neuroinflammatory mechanism of ITES. The significant elevation of IL-6/IL-8 in CSF, juxtaposed with the lack of diagnostic power of plasma IL-6 (AUC < 0.5), suggests that the inflammatory drive in ITES is compartmentalized within the CNS rather than being a mere spillover from systemic circulation. Finally, the blood metabolomics profile, primarily characterized by acylcarnitine accumulation, appears less optimal as a standalone diagnostic tool compared to urine but is crucial for elucidating the underlying mitochondrial dysfunction that predisposes children to this encephalopathy. Therefore, a tiered diagnostic strategy—utilizing urine for initial detection and CSF for confirmatory mechanistic diagnosis—may be the most pragmatic clinical application of our findings.

Several limitations warrant consideration. First, the sample size was relatively small, particularly for subgroup analyses, potentially limiting statistical power, while the retrospective, single-center design may introduce selection bias and constrain generalizability. Second, the heterogeneity of underlying infections and illness severity raises valid concerns that identified biomarkers might reflect general critical illness physiology—such as systemic inflammation, metabolic stress, or dextrose-containing IV fluids—rather than ITES-specific mechanisms. Indeed, the absence of a disease severity-matched control group (e.g., critically ill septic patients without encephalopathy) limits our ability to definitively distinguish ITES-specific signatures from epiphenomena of severe systemic infection. While sensitivity analyses stratifying by mRS scores and adjusting for key confounders (sepsis, hyperglycemia, seizures, intravenous fluids) demonstrated persistent associations between carnitine dysregulation/CSF cytokines and neurological severity, these adjustments cannot fully substitute for an appropriate comparative cohort. Third, the predictive model lacked validation in an independent cohort, posing a risk of overfitting. Although multiple comparisons were adjusted using the false discovery rate (FDR) method, the possibility of residual false-positive findings cannot be entirely ruled out. Fourth, while this study employed an integrated multi-omic approach (urine, blood, CSF), the findings remain exploratory. We have not systematically compared the diagnostic performance of single-matrix models (urine-only, blood-only, or CSF-only) versus combined models within a unified framework, nor have we quantified the independent contribution (e.g., AUC gain) of each biofluid to the overall discriminative power. Consequently, the optimal combination strategy and the marginal utility of integrating multiple matrices remain undefined.

Future studies should employ larger, prospective, multi-center cohorts with detailed phenotyping—including continuous metabolic monitoring and standardized nutritional support records—to further disentangle systemic illness from CNS-specific injury. Crucially, biomarkers require validation against disease severity-matched controls (e.g., non-encephalopathic septic patients) to establish specificity. Methodologically, future work should construct and compare single-source versus combined diagnostic models (e.g., urine + CSF), employing statistical methods such as Delong’s test to evaluate performance differences, and apply feature selection techniques (e.g., LASSO regression) to refine the current panel into a minimal yet optimal diagnostic combination for clinical translation.

## 5 Conclusion

In conclusion, this multi-omics study identified several potential biomarkers for ITES across various biological samples, including urine, blood, and CSF. Findings from urine samples highlight disruptions in glucose metabolism, whereas examination of blood metabolomics shows changes in acylcarnitine metabolism, reflecting pathophysiological processes underlying ITES. Additionally, the elevated levels of inflammatory cytokines, such as IL-6 and IL-8, in the CSF emphasize the role of immune responses and inflammation in disease progression. Through multi-omics analysis, this study initially identified a group of urine, blood and CSF biomarkers that have changed in children with ITES, suggesting the potential roles of glucose and lipid metabolism disorders as well as central nervous system inflammation in ITES. These findings lay the foundation for future validation of the diagnostic specificity of these biomarkers in larger-scale, more rigorous prospective studies, and for exploring their associations with disease severity and prognosis. Moreover, the therapeutic potential of immunomodulatory therapies, including anakinra and tocilizumab, warrants further exploration. The integrated approach of this study may provide a basis for further inquiry into the pathophysiology of ITES. With additional research, understanding these mechanisms might ultimately contribute to the development of novel strategies aimed at improving patient care.

## Data Availability

The raw data supporting the conclusions of this article will be made available by the authors, without undue reservation.
