# Supplementary material for: Pediatric infection-triggered encephalopathy syndromes: a multidimensional biomarker analysis
Source: Front Neurol. 2026 Mar 18;17:1730057. doi: 10.3389/fneur.2026.1730057 (PMC13038577; doi:10.3389/fneur.2026.1730057)
Supplement: Supplementary file 1 [file Supplementary_file_1.docx]

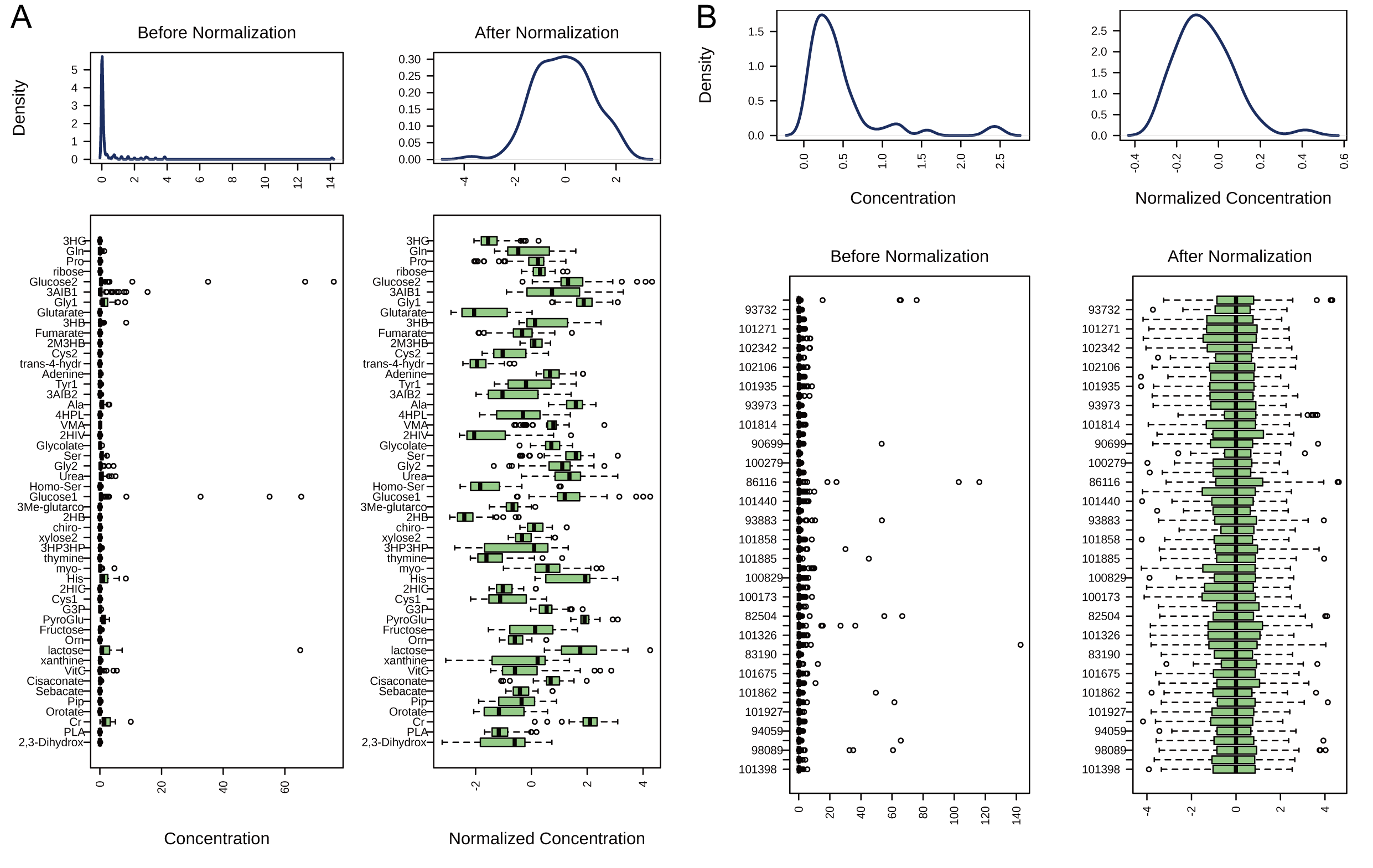


**Figure S1. Urine Metabolite Data Normalization and Transformation.** (A) Sample normalized by median. (B) Data Transformation: Log Transformation (Base10).


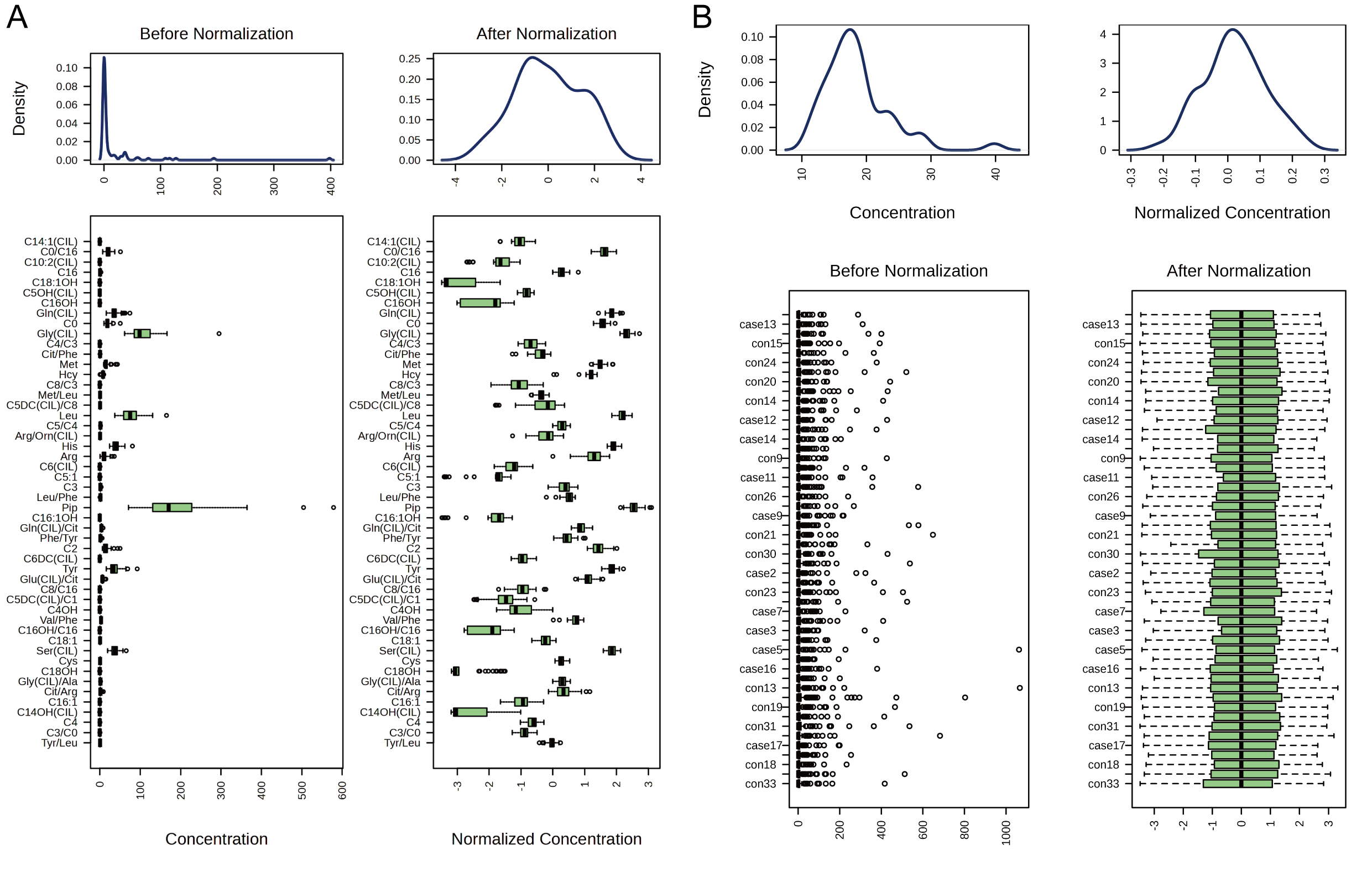


**Figure S2. Blood Metabolite Data Normalization and Transformation.** (A) Sample normalized by median. (B) Data Transformation: Log Transformation (Base10).


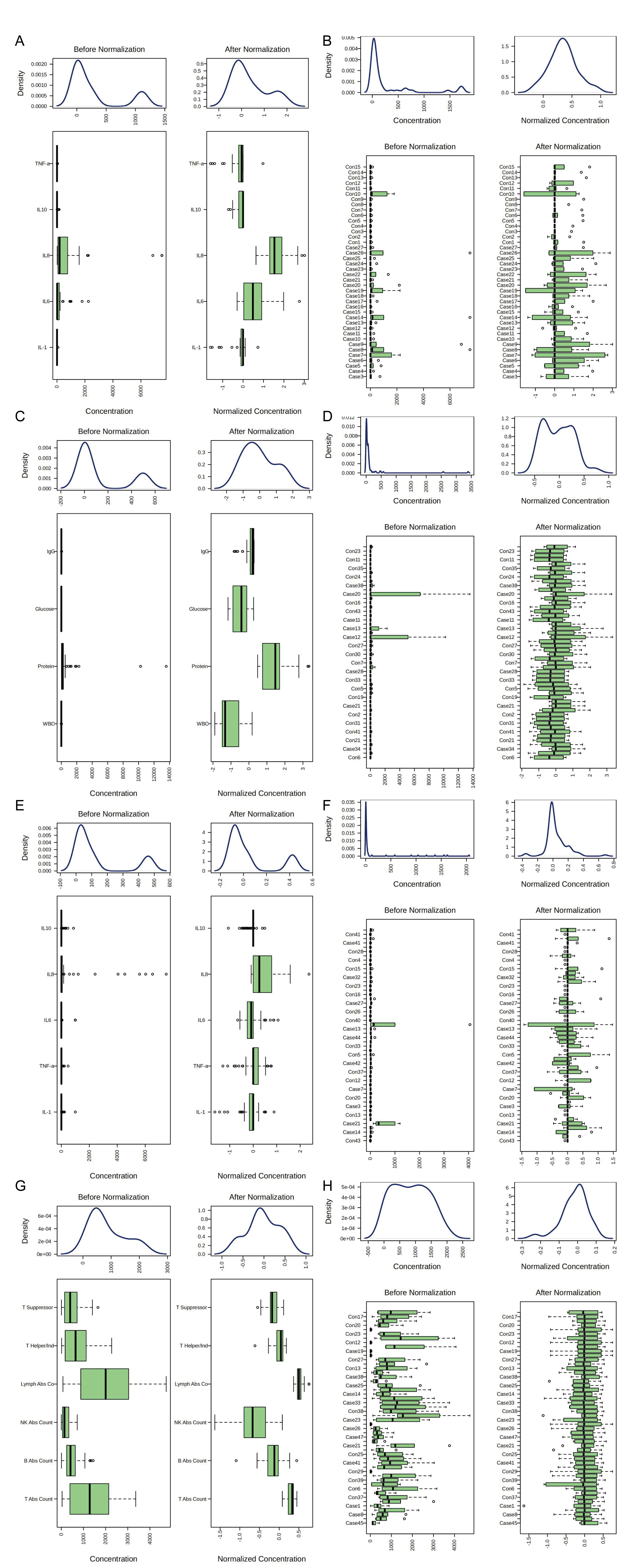


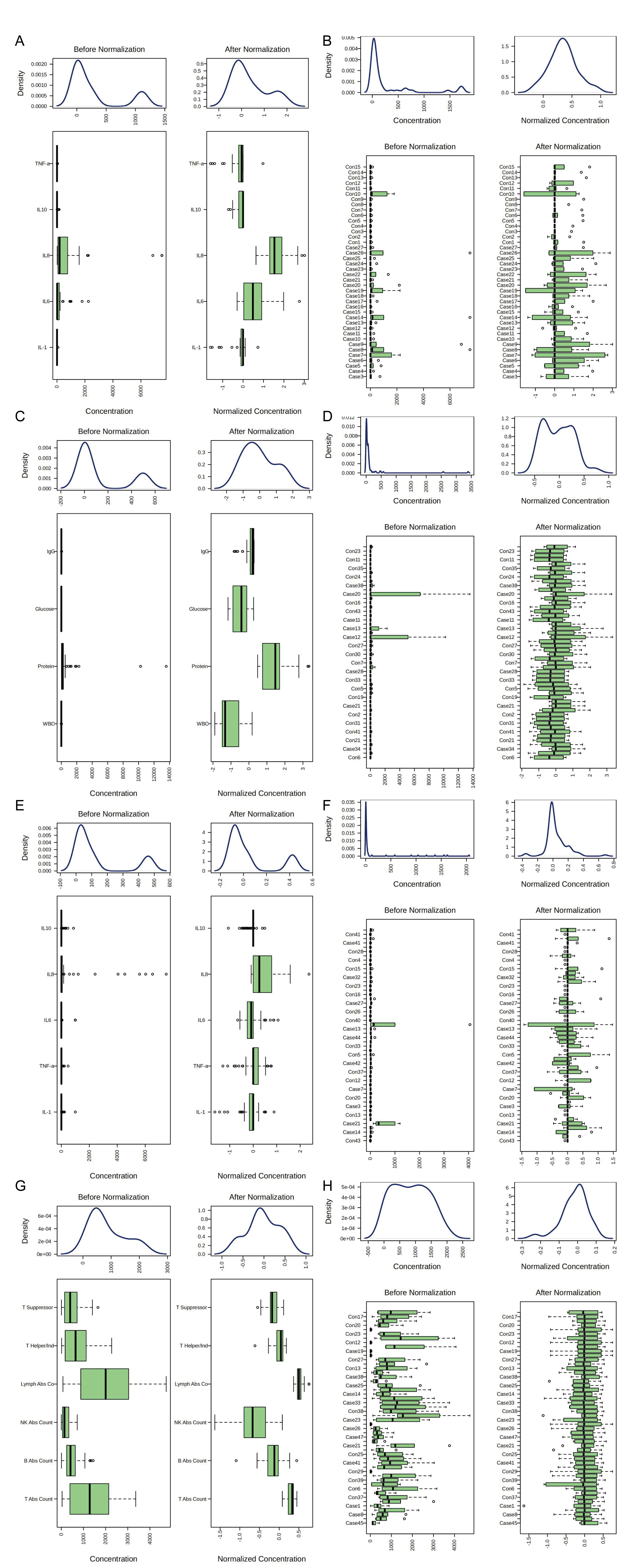


**Figure S3. Median Normalization and Log-Transformation of blood cytokines, blood immune cells, CSF cytokines, and CSF routine and biochemical.** Median normalization applied to: (A) CSF cytokines, (C) CSF biochemical parameters, (E) Blood cytokines, (G) Blood immune cells. Log_10_-transformation applied to: (B) CSF cytokines, (D) CSF biochemical parameters, (F) Blood cytokines, (H) Blood immune cells.

**
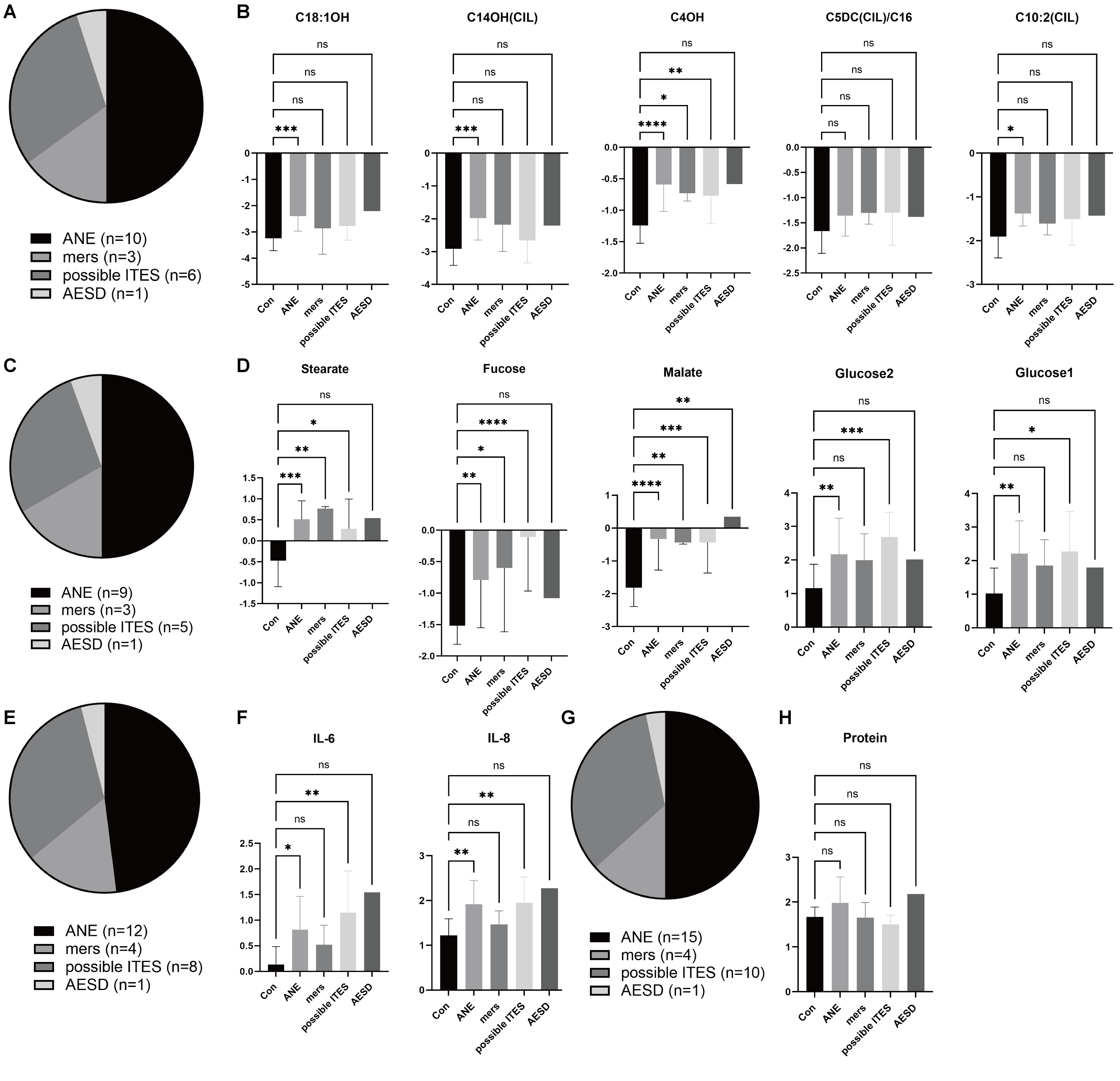
**

**Figure S4. Characterization of ITES subcomponents and their biomarker expression.** The compositional fractions of ITES subcomponents in blood (A), urine (C), CSF cytokines (E), and CSF protein (G) are shown in pie charts. The expression levels of corresponding biomarkers, including blood metabolites (B), urinary metabolites (D), CSF cytokines (F), and CSF protein (H), are displayed as bar graphs. Data are presented as mean ± SD. Statistical differences were analyzed by one-way ANOVA. P < 0.05, P < 0.01, P < 0.001, P < 0.0001.

Metabolite identification

| Compound abbreviation | The full English name of the compound |
| --- | --- |
| 3HB | 3-Hydroxybutyric acid |
| 3HP3HP | 3-Hydroxybenzoic acid-3-hydroxypropionic acid |
| C4OH | Hydroxybutyryl carnitine |
| C14OH | Hydroxytetradecanoyl carnitine |
| C18:1OH | Hydroxyoctadecenoyl carnitine |
| C5DC/C16 | Hydroxyoctadecenoyl carnitine / Hexadecanoyl carnitine |
| C10:2 | Decadienoyl carnitine |
| CIL | Reagent manufacturer |
